# Initiation of feeding by four sympatric Neotropical primates (*Ateles belzebuth, Lagothrix lagotricha poeppigii*, *Plecturocebus (Callicebus) discolor,* and *Pithecia aequatorialis*) in Amazonian Ecuador: Relationships to photic and ecological factors

**DOI:** 10.1371/journal.pone.0210494

**Published:** 2019-01-23

**Authors:** D. Max Snodderly, Kelsey M. Ellis, Sarina R. Lieberman, Andrés Link, Eduardo Fernandez-Duque, Anthony Di Fiore

**Affiliations:** 1 Department of Neuroscience, University of Texas at Austin, Austin, TX, United States of America; 2 Department of Anthropology, University of Texas at Austin, Austin, TX, United States of America; 3 Department of Biological Sciences and School of Management, Universidad de Los Andes, Bogota, Colombia; 4 Department of Anthropology, Yale University, New Haven, CT, United States of America; Fred Hutchinson Cancer Research Center, UNITED STATES

## Abstract

We examined photic and ecological factors related to initiation of feeding by four sympatric primates in the rain forest of Amazonian Ecuador. With rare exceptions, morning activities of all taxa began only after the onset of nautical twilight, which occurred 47–48 min before sunrise. The larger spider and woolly monkeys, *Ateles belzebuth* and *Lagothrix lagotricha poeppigii*, left their sleeping trees before sunrise about half the time, while the smaller sakis and titi monkeys, *Pithecia aequatorialis* and *Plecturocebus (formerly Callicebus) discolor*, did not emerge until sunrise or later. None of the four taxa routinely began feeding before sunrise. *Pithecia* began feeding a median 2.17 h after sunrise, at least 0.8 h later than the median feeding times of the other three taxa. The early movement of *Ateles* and *Lagothrix*, and late initiation of feeding by *Pithecia* are consistent with temporal niche partitioning. Among most New World primate species, all males and many females, have dichromatic color vision, with only two cone photopigments, while some females are trichromats with three cone photopigments. Current evidence indicates that the dichromats have a foraging advantage in dim light, which could facilitate utilization of twilight periods and contribute to temporal niche partitioning. However, in our study, dichromatic males did not differentially exploit the dim light of twilight, and times of first feeding bouts of female *Ateles* and *Lagothrix* were similar to those of males. First feeding bouts followed a seasonal pattern, occurring latest in May-August, when ripe fruit abundance and ambient temperature were both relatively low. The most frugivorous taxon, *Ateles*, exhibited the greatest seasonality, initiating feeding 1.4 h later in May-August than in January-April. This pattern may imply a strategy of conserving energy when ripe fruit is scarcer, but starting earlier to compete successfully when fruit is more abundant. Lower temperatures were associated with later feeding of *Ateles* (by 26 min / °C) and perhaps *Pithecia*, but not *Lagothrix* or *Plecturocebus*. The potential for modification of temporal activity patterns and temporal niche partitioning by relatively small changes in temperature should be considered when predicting the effects of climate change.

## Introduction

New World monkeys represent a diverse radiation of primates that have spread to occupy a variety of ecological niches. Sympatric species inhabiting the same forest environments have developed a variety of behavioral adaptations that allow them to coexist through niche partitioning [1]. For diurnal monkeys, these behavioral adaptations include use of different microhabitats [2], different locomotor and foraging behaviors [3, 4], consumption of different plant parts or species [5], and the use of different fallback resources during periods of fruit scarcity [6, 7].

Another potential mechanism of coexistence is temporal partitioning of activities across the day [8]. Most accounts of temporal partitioning compare nocturnal animals with sympatric diurnal ones [9], but diverse taxa also partition the day (e.g. lizards [10], dung beetles [11]) or the night (e.g. bats [12]) by being active at different times. However, temporal partitioning by subdividing the day seems to have received little attention among primatologists.

Here, we consider the extent to which niche partitioning among a sympatric group of diurnal primates includes a temporal component, as evidenced by the time when individuals begin to forage and to feed. We also consider how the timing of these behaviors is related to three ecological factors: temperature, rainfall, and habitat-wide abundance of ripe fruit.

One possible contributor to temporal niche partitioning is the diversity of color vision systems of New World monkeys and its influence on foraging success in light conditions occurring at different times of day. With the exception of howler monkeys (*Alouatta* spp.), all male New World monkeys and many females have dichromatic color vision, with only two cone photopigments in their retinas, one S pigment that is maximally sensitive to short wavelengths, and one M/L pigment maximally sensitive to mid-to-long wavelengths [13, 14]. These dichromats have poor color discrimination at longer wavelengths, where many ripening fruits reflect light, a situation traditionally considered to be a handicap [15]. However, if females acquire two different M/L gene alleles, one on each X chromosome, they can achieve trichromacy with three cone photopigments and acute color discrimination throughout the visible spectrum.

A foraging advantage for trichromatic monkeys over dichromats consistent with a benefit from better color discrimination [15], has been demonstrated both experimentally and in the field [16–20]. In addition, a possible group-level benefit of trichromacy has been identified in a strepsirrhine primate with polymorphic trichromacy similar to that seen in New Word monkeys by comparing groups that include trichromats, with groups composed only of dichromats [21].

In contrast, there are situations in which dichromats have been shown to have an advantage. For example, dichromatic *Saguinus* caught a greater proportion of camouflaged insects both in captivity and in the field than their trichromatic group-mates [22], consistent with an ability of dichromats to break camouflage [23]. Also, captive experiments with marmosets [24], and field studies of capuchins [25, 26], have provided evidence of a dichromat advantage when foraging in dim light. For the capuchins, the dichromat advantage was greatest when animals were foraging for camouflaged insects [25]. A recent study of humans also found that dichromats have better achromatic contrast sensitivity than trichromats [27], which might be helpful at light levels that are suboptimal for color discrimination.

These results raise the possibility that individual dichromats, and species with large numbers of dichromats, might systematically exploit dim light conditions, including twilight, to their advantage. Consistent with this idea, a comparative study found that a completely dichromatic species, *Lemur catta*, foraged in lower light levels than several polymorphic species, including sympatric *Propithecus v*. *verreauxi* [28]. However, temporal factors, such the progression from dim to bright light as the day advances from night through twilight to sunrise, were not considered.

To evaluate the hypothesis that diurnal New World primates may occupy different temporal niches with respect to the daily solar cycle, we analyzed morning activities of four sympatric species in the lowland rain forest of Amazonian Ecuador. The study species include two highly frugivorous taxa, (spider monkeys, *Ateles belzebuth*, and woolly monkeys, *Lagothrix lagotricha poeppigii*) that are important seed dispersers [29, 30], a genus with a more diverse diet (titi monkeys: *Plecturocebus discolor*, formerly known as *Callicebus discolor*) [31], and a seed predator (saki monkeys: *Pithecia aequatorialis*) [32]. The home ranges of these taxa vary widely in average area (e.g., *Pithecia*, 57 ha, 6 social groups [33]; *Ateles* 632 ha, 1 group [34]). However, they are spatially superimposed such that the smaller home ranges of *Plecturocebus* and *Pithecia* are contained within the larger home ranges of *Lagothrix* and *Ateles*; there is no obvious spatial separation of the taxa [35].

The period around sunrise should be favorable for detecting differences in activity patterns among species related to light levels and to temporal niche partitioning. At this time, all taxa experience a wide range of light levels as the sun rises, and individuals of all taxa have likely been without food for many hours. To identify the earliest components of their activity patterns, we have analyzed times relative to sunrise when monkeys showed signs of awakening by urinating and defecating, when the monkeys left their sleeping trees and began to forage, and when they had their first feeding bout.

## Methods

### Study site

Data were collected in the context of a multispecies primate research program started at the Tiputini Biodiversity Station in 2003. The station is located immediately adjacent to the 900,000 ha Yasuní National Park [36] in eastern Ecuador (76°08´W, 0°38´S) and the site covers ~640 protected ha of primary lowland evergreen rainforest (elevation 196–269 meters above sea level). Ninety percent of the area is terra firme (unflooded) forest, bordered by narrow areas of riparian varzea that flood during times of heavy rainfall upriver from the site.

**Fig 1:**
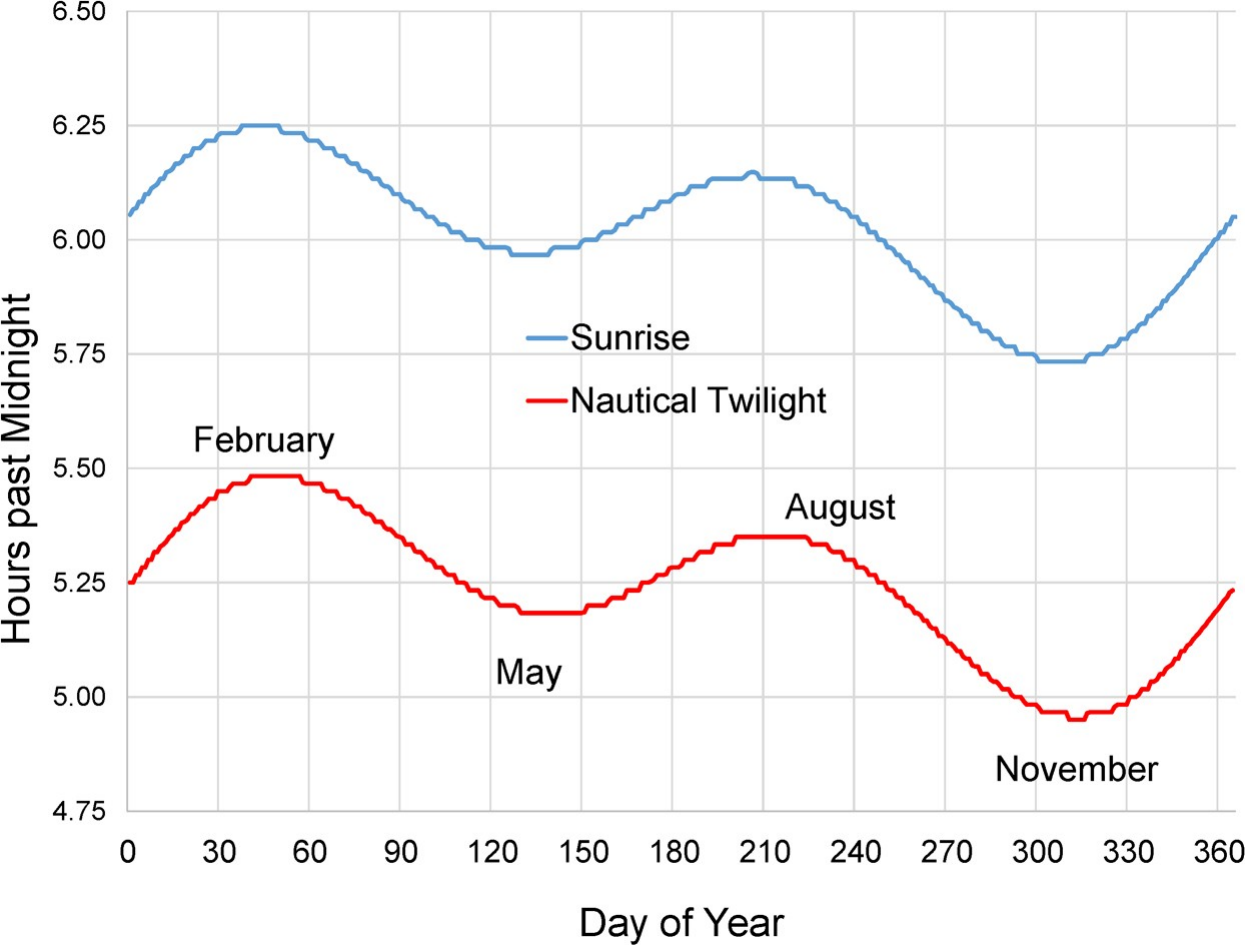
Annual cycle of sunrise and nautical twilight at Tiputini. Sunrise, the time when the edge of the sun first appears above the horizon, and nautical twilight, the time when the sun is 12º below the horizon, at the Tiputini Biodiversity Station (76°08’ W, 0°38’ S). Day 1 is January 1. Values were downloaded from the website of the US Naval Observatory (http://www.usno.navy.mil/USNO/astronomical-applications); they vary only 1–2 min from year to year. Curves are averages for years 2006–2013.

At this latitude near the equator, photoperiod is virtually constant throughout the year, but the time of sunrise varies over a range of approximately 30 min during the annual cycle (Fig 1). To follow standard practice in circadian biology and to facilitate comparisons with study sites at different latitudes, all behavioral data are referenced to sunrise and to nautical twilight, which occurs 47 to 48 minutes prior to sunrise. “Nautical” twilight refers to the time when a mariner at sea can first discern the horizon; it is also the time when a human observer at the top of the canopy can first visualize the horizon.

### Temperature and rainfall

**Fig 2:**
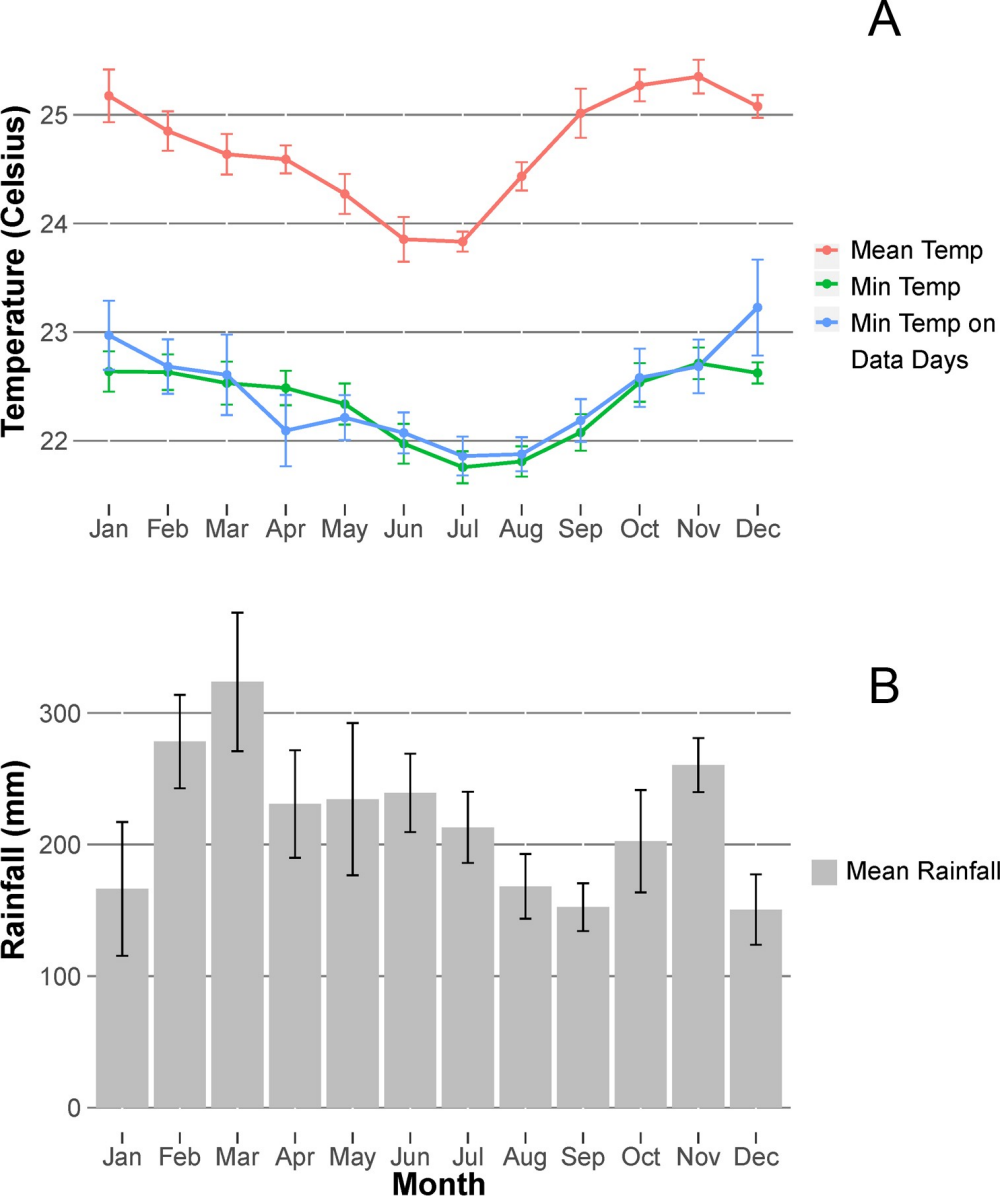
Weather patterns at the Tiputini Biodiversity Station. **A**. Monthly variation in mean daily temperatures, mean daily minimum temperatures, and mean daily minimum temperatures for the subset of days when behavioral data were collected. Temperature was recorded for 77% of 4364 days between June 2005 and June 2017. **B.** Mean monthly rainfall recorded for 86% of 2871 days between June 2008 and April 2016. Months with data for fewer than 15 days were excluded from the averages. Error bars indicate standard errors.

Temperature was recorded hourly by automated data loggers (Marathon EZ Logger; Kestrel Drop D2) placed in a shaded, open hallway at the research station (Fig 2A). We report three different measures of temperature, calculated on a monthly basis: 1) Mean daily temperature, for comparison with other study sites; 2) Mean daily minimum temperature, because it usually occurs just before sunrise, around the time when monkeys may first become active, and 3) Mean daily minimum temperatures calculated only for days when monkey behavioral data were obtained, to confirm that the data were obtained under representative conditions. On a monthly basis, mean daily temperature varied only from 23.8–25.4º C. The daily minimum temperature showed even less variation, with monthly mean values ranging only from 21.8–22.7 º C. The lowest temperatures occurred in June, July, and August.

Rain was collected using a manual gauge, and 24-h rainfall was recorded each evening sometime between 18 and 20h. Although there is not a pronounced dry season at the site, mean monthly rainfall varied by about a factor of two over the course of the year (Fig 2B). A similar annual pattern, based on a longer time period from 1982 to 2012, has been reported for the city of Puerto Francisco de Orellana (also known as El Coca), about 94 kilometers WNW of the research station (76°59´W, 0°28´S, https://en.climate-data.org/location/2975/).

On days when behavioral data were collected, mean temperature was negatively correlated with 24-hr rainfall (r = -0.29, p <0.0001, df = 331), whereas the minimum temperature was not (r = 0.002, p = 0.97, df = 331). This pattern probably reflects the fact that showers often occurred in the afternoon, lowering the mean temperature for the day without affecting the minimum temperature, which occurred in the early morning hours.

### Phenological data and estimates of ripe fruit abundance

**Fig 3:**
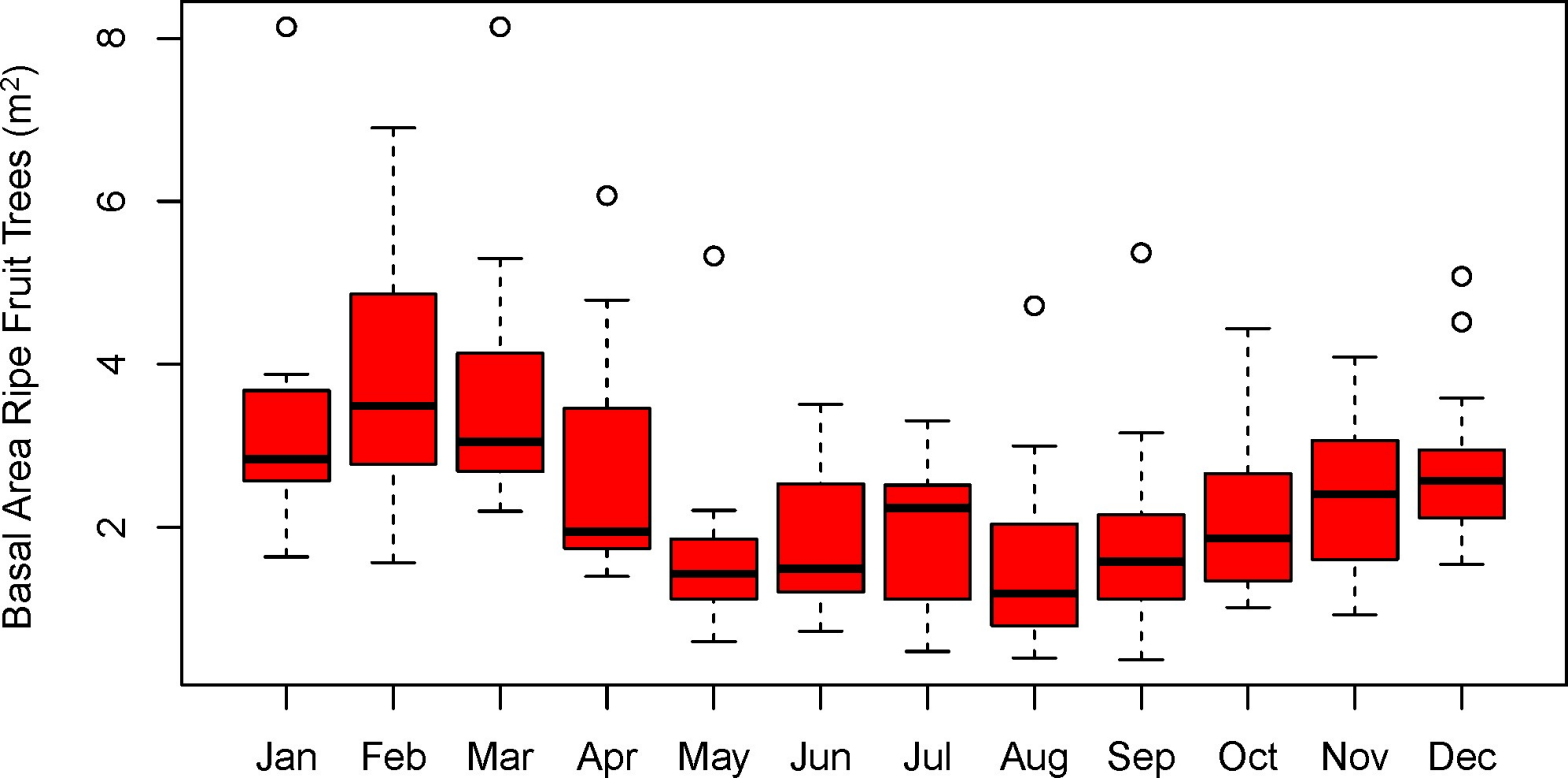
Ripe fruit abundance at Tiputini. Monthly abundance of ripe fleshy fruit as estimated by total basal area of trees bearing ripe fruit (m^2^) along a 9 km phenology transect. Values can be converted to basal area per hectare by dividing by 5.25 ha, which is the total sampling area of the transect.

Phenological data were collected twice a month for 79% of 252 biweekly sampling periods between August 2006 and February 2017. Any tree bearing ripe fleshy fruit (hereafter simply called “ripe fruit”), or supporting a liana producing ripe fruit, and whose crown overhung a 9 km long set of ‘fruit transects’ was identified to the species or morphospecies level. Judgements of ripeness were based on our research team’s knowledge of the typical ripening pattern of the fruit gleaned from more than 10 years of observation, not just based on fruit color. During each twice monthly review, the perpendicular distance from the fruiting tree to the transect was measured, as well as the circumference of the tree trunk at a height of 1.3 m. The average distance of the fruit trees from the transect was taken as one-half the effective width of the sampled area, which resulted in a transect with an effective sampled width of about 5.8 m and a total monitored area of 5.25 ha. Tree diameters at breast height (DBH) were calculated (as circumference/π), assuming a circular cross-section, and the basal area of each tree was calculated as the area of this cross-section. The basal area was then used as an index of the productivity of the tree or of associated lianas [37]. Because trees have fruiting seasons that cover more than one sampling period, we allocated a proportion of the productivity to each of the sampling periods according to the coefficients of Pascal´s triangle [38, 39]. For example, if a tree produced fruit in four successive periods, its fruit crop was distributed among those four periods in the proportions of 1:3:3:1. The proportion of the basal area of all trees with ripe fruit along the transect during each sampling period was summed to obtain an indicator of habitat-wide ripe fruit abundance at the study site (Fig 3).

### Behavioral data

Behavioral data have been collected since 2002 as part of Proyecto Primates, a research program on the comparative socioecology of Neotropical primates [40–45]. We present analyses of data collected by 50 trained observers between 2005 and 2016 using a combination of focal animal sampling, group scan sampling interspersed with the time points of the focal samples, and *ad libitum* observations [46]. Most observers focused primarily on one or two primate species and collected only small amounts of data on other species, so it is not possible to completely control for inter-observer differences. However, given the large number of observers, potential observer biases are expected to distribute across study species to lessen their effects on the comparisons. More than 90% of the data were derived from observations of one large troop of *Ateles* (MQ-1, which ranged in size from 25–37 individuals), three multi-male, multi-female groups of *Lagothrix* (C, G, and D, with 12–30 individuals/group), three groups of *Plecturocebus* (formerly *Callicebus*) (B, K, and L, with 2–6 individuals/group), and three groups of *Pithecia* (M, P, and S, with 3–9 individuals/group). The remaining data were derived from opportunistic observations of individuals from neighboring groups or from individuals that were not identified.

### Movement from the sleeping site

Movement out of a sleeping tree initiates the first foraging activity, as individuals explore their surroundings and/or search for a feeding tree. Because the larger taxa sometimes leave the sleeping tree quite early, it can be challenging for observers to establish contact before individuals leave the sleeping tree, creating a potential bias due to missing some of the earliest movements for these taxa.

We attempted to minimize bias by analyzing the data in two steps. First, data were included only when they met the following criteria: 1) the sleeping site was known from the previous evening's records, 2) the observer encountered the monkeys in the same site on the day of observation, and the monkeys were inactive at the time of encounter, and 3) the observer recorded the specific time of the first movement from a sleeping tree by any member of the group.

Applying these stringent criteria resulted in a sample of 116 observations of movement from the sleeping tree. However, sometimes observers knew where the monkeys went to sleep one evening, but when they arrived at the sleeping site the next morning, the monkeys were already moving or foraging out of the sleeping tree, still within sight of the observer. We define these cases as “near misses” and use the times the monkeys were encountered as best estimates of first movements, while acknowledging that these times are slightly later than the true values. Applying this criterion added to the sample eight cases for *Ateles*, one for *Lagothrix*, and none for the other taxa. Including the near misses shifted the median time of first movements 11–12 min earlier for *Ateles*, resulting a more realistic comparison with the other taxa, because it partially corrected for missing the earliest movements. The combined sample of 125 observations was used to analyze first movements from the sleeping site.

Because movement was continuously recorded only in a subset of the behavioral sampling protocols used during the entire study period, the sample size for first movements from the sleeping tree is much smaller than sample size for first feeding bouts (described below).

In several protocols, times of first defecation and urination were recorded continuously, and 131 cases were recorded across the four taxa.

### First feeding bouts

Protocols for behavioral sampling from all four taxa included continuous recording of feeding bouts, which were defined as 5 “monkey-minutes” of cumulative feeding by the members of a group (*e*.*g*., one monkey feeding for five minutes, or five monkeys for one minute, both satisfied this criterion). This criterion was intended to distinguish casual consumption of a food item, such as a fruit picked and eaten *en route* during travel, from more sustained consumption of a food source. It has the advantage of treating all taxa equally, but it does not attempt to adjust for differences in feeding patterns or total intake.

Similar to the analysis of first movement from the sleeping tree, we used multiple inclusion criteria. In the first step, data were only considered if monkeys were encountered in a sleeping tree and then followed to the site of the first feeding bout, which resulted in a sample of 396 bouts. To these data, we added eight “near miss” cases (as described above) when the monkeys were first encountered within sight of the sleeping tree. Finally, we added 24 cases of “early encounters,” where the sleeping tree had not been securely identified the night before, but the monkeys were encountered at least 0.3 hours before sunrise and they were still inactive at the time the observer arrived at the group location, which suggests that they were still in the sleeping trees. Adding these near misses and early encounters shifted the median time for first feeding bouts of *Ateles* 12.6 min earlier, while medians for all other taxa remained stable to within 1–2 min. This combination of criteria resulted in a total sample of 428 first feeding bouts. Even with these additions, estimated times of *Ateles*’ behaviors are undoubtedly slightly later than the true values because some of the earliest instances were missed, with the result that differences between *Ateles* and the other taxa are likely underestimated.

### Delay and distance between the sleeping tree and the first feeding bout

For this analysis, we only included records based on the same group on the same day that also had complete ecological data for temperature, rainfall and ripe fruit abundance. We calculated the feeding delay as the time of the first feeding bout minus the time of first movement from the sleeping site. For a subset of the data, we calculated the distance traveled from the sleeping site to the first feeding tree, based on GPS coordinates recorded by the observers.

### Human visual observations

During three field seasons (Nov 2012-Jan 2013, Jun 2013-Feb 2014, Jul-Aug 2015), observers with clinically normal vision recorded the times at which important features of the forest canopy became visible as the sun rose, starting before nautical twilight and continuing until after sunrise. The human observations were intended to identify visual information that was potentially available to the monkeys and could influence their early activities, given the similarities of the visual systems of diurnal monkeys and humans [47–53]. Two observers viewed the canopy from a platform at a height of 32 meters, comparable to the maximum height where monkeys slept and foraged. From one to three other observers stood on the forest floor monitoring the morning wakeup of the monkeys. On 37 days, simultaneous observations were made at both canopy and ground levels; when staffing was limited, additional observations were made in only one location, canopy or ground (numbers reported in Results). All observers recorded the earliest time at which they could see 1) large branches typical of ones that monkeys use to move about the canopy, 2) small terminal tree branches in the canopy, and 3) colors of objects in the canopy or of a color calibration chart (DGK Color Tools) held in their hand. If the monkeys—at least those females who are trichromats—had visual capacities similar to humans, we reasoned that these qualitative judgments should correspond to 1) the time that monkeys could begin to move about the canopy, 2) the time the monkeys could first visualize the forms of small food objects, and 3) the time that color would become useful for detecting fruit and judging its ripeness. Dichromatic monkeys might be able to visualize forms even earlier. Human observers also noted whether there was sufficient moonlight to enhance vision during morning nautical twilight.

### Data analyses

We analyzed statistical differences in times of behavioral events with multivariate linear models in R, version 3.3.0 [54]. Analyses were based on linear mixed models using the *lmer* function from the package "lme4" version 1.12. The factors included in the initial full models and the statistical significance tests performed are summarized in Table 1. The dataset used for the study is included as S1 Table.

**Table 1:** Statistical models, factors and interactions, and tests performed.

Response Variable and Statistical Tests	Fixed Factors and Interactions among Predictor Variables	Random Factors
1) Time to depart sleeping tree2) Time of first feeding bout3) Delay between departure from sleeping tree and feedingTest differences between taxa	Taxon	Month
	Temperature	Year
	Rainfall	Social Group or Animal ID
	Ripe Fruit Abundance	
4–7) Time to depart sleeping tree, one model for each taxon8-11) Time of first feeding bout, one model for each taxonTest for ecological influences	Temperature	Month
	Rainfall	Year
	Ripe Fruit Abundance	Social Group or Animal ID
12) Time of first feeding boutin relation to Ripe Fruit AbundanceTest differences between taxa	Taxon* Ripe Fruit Abundance	Month
	Temperature	Year
	Rainfall	Social Group or Animal ID
13) Time of *Ateles* first feeding bout14) Time of *Lagothrix* first feeding boutTest differences between males and females	Sex	Month
	Temperature	Year
	Rainfall	Animal ID
15) Seasonal variation in times of first feeding boutsTest differences between taxa and between quadrimesters	Taxon*Quadrimester	Year
	Temperature	Social Group or Animal ID
	Rainfall	
	Ripe Fruit Abundance	

*Temperature*: minimum temperature of the day (which occurred in the pre-dawn hours, °C). *Rainfall*: square root (to reduce skew) of the 24 hr accumulation (mm) starting the evening before. *Ripe fruit abundance*: Basal area of trees bearing ripe fruit in m^2^ per hectare, measured twice a month. *Quadrimesters*: 4-month blocks, corresponding to January-March, April-July, and August-December. Social Group or Animal ID: social group identity for *Plecturocebus* and *Pithecia*, individual animal identity for *Ateles* and *Lagothrix*.

We constructed models both with raw data and with transformed times (square root of (data +1) to accomodate negative numbers). To maximize statistical power, we removed random factors (month, year, social group, or animal ID) that did not have statistically significant effects from the initial full models, using conservative criteria (p≥0.2); the adequacy of the reduced models was confirmed by likelihood ratio tests showing that the reduced models were not significantly different from the full models (p>0.5 in one case, all others p>0.8). The random factors included in the final models are tabulated in S2 Table. We estimated statistical significance of mean differences using the *lsmeans* function, version 2.25. All reported p values are for two-tailed tests, with the Tukey adjustment for multiple comparisons, except for tests of the effects of rainfall, where we report one-tailed p-values because we predicted that rainfall would delay activities.

Histograms of the residuals of the models were inspected to confirm that distributions of the residuals approximated normal distributions. We used the square root of rainfall values in all models to reduce skewness of the residuals. The ratio of the skewness of the residuals to its standard error (skewness ratio) was calculated as an indication of the deviation from a normal distribution. Results of significance tests are reported for the data format (raw or transformed times of behavioral events) with the lower skewness ratio, indicating that the residuals more closely approximated a normal distribution. Sixty percent of the skewness ratios were less than 1.3, and 40% were between 2.1 and 3.0. For calculation of regression coefficients in models 7–10, we also report results from models based on raw data even if the residuals were more skewed, because the results are in intuitive, useful units. For these exceptional cases, the skewness ratios were still less than 4.0. We confirmed that models had minimal collinearity (all variance inflation factors (vif) less than 2).

For this long-term study of wild primates, a small number of subjects of each species were captured via remote anesthetization for fitting with radiocollars to permit regular localization for observation. Anesthetization was performed by darting the animals intramuscularly using a DanInject CO2-powered rifle and PneuDart commercial darts (type P, volume sizes 0.5, 1.0, 1.5, and 2.0 cc) filled with an appropriate dosage of either ZolatilTM (tiletmine/zolazepam: 12–18 mg/kg body weight) or ketamine HCl (~25–50 mg/kg body weight), based on published recommendations and consultation with both wildlife and lab-based veterinarians. Remote anesthetization and capture protocols, as well as health monitoring procedures and strategies for dealing with risks associated with capture, were developed in consultation with veterinarians from New York University, the University of Texas at Austin, and the Universidad San Francisco de Quito in Ecuador. All animal protocols were approved by the Institutional Animal Care and Use Committee/University Animal Welfare Committee of New York University, the University of Texas at Austin, and/or Yale University. Currently active IACUC protocol numbers from the University of Texas include AUP-2017-00039, AUP-2017-00259, and AUP-2017-00260.

This research was conducted on protected land and complied with all Ecuadorian legislation. Research approvals were obtained from the Ministerio de Ambiente of Ecuador and from the scientific advisory committee of the Tiputini Biodiversity Station, Universidad San Francisco de Quito.

## Results

### Movement from the sleeping tree

**Fig 4:**
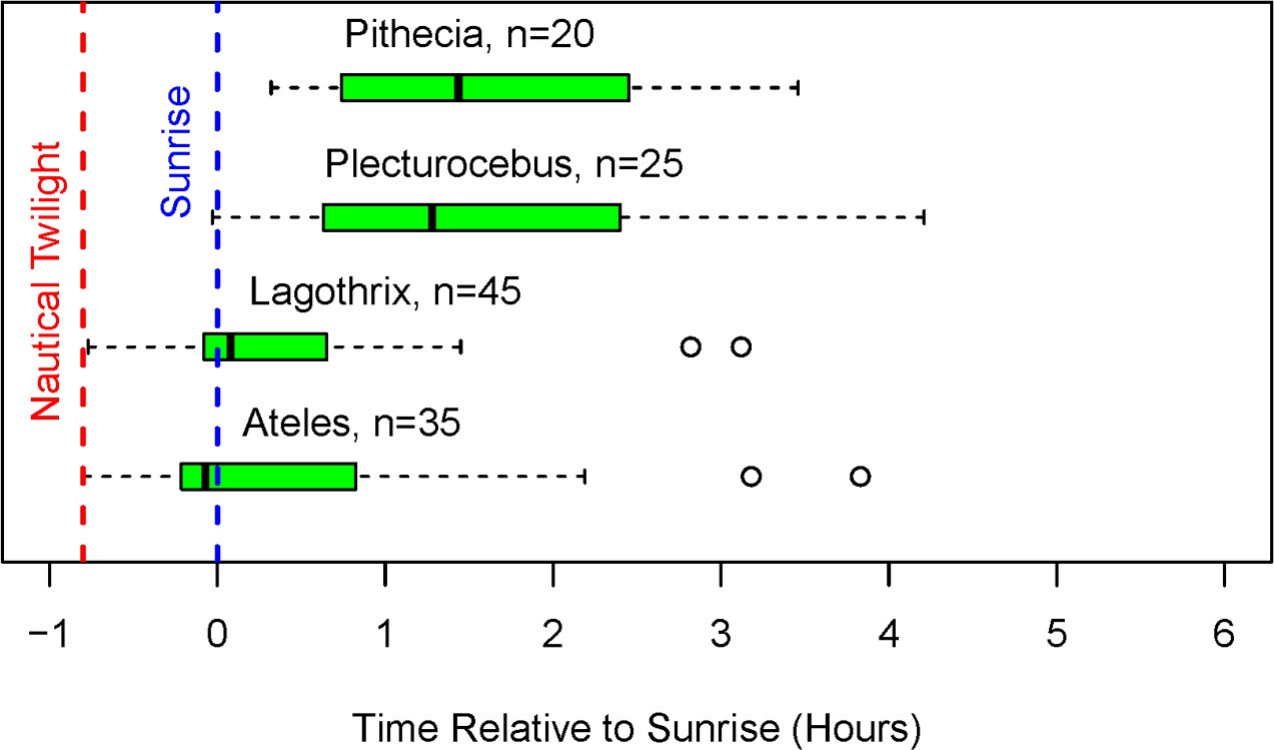
Time of first movement from the sleeping tree (hours relative to sunrise). Boxplots: median and interquartile range (IQR); whiskers indicate extreme values unless there are data points more than 1.5*IQR below the lower quartile or more than 1.5*IQR above the upper quartile, in which case whiskers stop at the first data points within those boundaries and open circles indicate more extreme points. n = number of observations.

With rare exceptions, individuals of all taxa slept in trees that were not contemporaneously used for feeding. *Lagothrix*, *Plecturocebus*, and *Pithecia* never slept in a tree that was also used for feeding, and *Ateles* slept in a feeding tree on only 5 occasions, all in the same large *Brosimum lactescens*. Once individuals awakened, they often defecated and urinated before moving away from the sleeping tree and beginning to forage. For the larger monkeys, more than half the recorded defecations and urinations occurred before sunrise (*Ateles*, 51%, n = 67; *Lagothrix*, 57%, n = 37) and one defecation/urination event for both taxa occurred before nautical twilight. The smaller monkeys, *Plecturocebus* (n = 15) and *Pithecia* (n = 12) awoke later and all but one defecation/urination event occurred after sunrise. The earlier awakening signaled by defecation and urination of the larger monkeys was followed by earlier departure times from the sleeping trees (Fig 4).

We have no records of monkeys of any species leaving the sleeping tree before the beginning of nautical twilight. On eight occasions, *Lagothrix* was encountered in the sleeping tree at least 10 min before nautical twilight, but the monkeys did not leave the sleeping tree until after nautical twilight. This outcome is consistent with the idea that poor visibility of tree limbs in the canopy hinders locomotion before nautical twilight.

There were striking differences in activity patterns among taxa during the transition from nautical twilight to sunrise. The larger monkeys, *Ateles* and *Lagothrix*, emerged from their sleeping trees before sunrise about half the time. In contrast, the smaller monkeys, *Pithecia* and *Plecturocebus*, did not leave their sleeping trees until sunrise or later. Indeed, the median and the mean times of departure from the sleeping tree for the small monkeys were more than an hour later than the corresponding times for the large monkeys. The later values for the smaller monkeys are not due to failure to encounter the monkeys early, because observers were with the groups before sunrise for about half of the observations (*Pithecia*, n = 9; *Plecturocebus*, n = 13), and on none of those occasions did the monkeys leave their sleeping trees before sunrise.

Differences between taxa were tested statistically with a mixed model using transformed times of departure from the sleeping tree (Table 1, Model 1) that reduced to a linear model accounting for 48% of the variance in departure time. The model is based on the subset of total observations that included data for all three ecological variables (temperature, rainfall, and ripe fruit abundance). Table 2 illustrates that the means and medians of the subset of samples with complete ecological data were representative of the total sample. The large monkeys, *Ateles* and *Lagothrix* both departed their sleeping trees more than an hour earlier than the smaller monkeys, *Plecturocebus* and *Pithecia* (p<0.001); differences between the two large taxa, *Ateles* and *Lagothrix* (p = 0.14), and between the two small taxa, *Plecturocebus* and *Pithecia* (p = 0.47), were not statistically significant.

**Table 2:** Time of first movement from the sleeping tree (hours relative to sunrise).

Taxon	All observations (n)	Median (IQR)	Mean ±SE	Ecological subset (n)	Median (IQR)	Mean ±SE
*Pithecia*	20	1.44 (1.51)	1.59 ± 0.22	18	1.63 (1.66)	1.66 ± 0.23
*Plecturocebus*	25	1.28 (1.77)	1.44 ± 0.22	23	1.28 (1.79)	1.43 ± 0.24
*Lagothrix*	45	0.08 (0.73)	0.37 ± 0.11	24	0.22 (0.84)	0.55 ± 0.18
*Ateles*	35	-0.07 (1.04)	0.42 ± 0.19	20	-0.13 (0.29)	-0.04 ± 0.13

Left columns: Number of observations, medians, and means for the the total sample. Right columns: Medians and means for the subset of observations that included data for all three ecological variables—temperature, rainfall, and ripe fruit abundance.

#### Human visual observations

Human observers on a platform in the canopy recorded the earliest times when features of the environment relevant to the monkeys’ behavior became visible (Table 3). Large tree branches became visible a median 27 min before sunrise and small terminal branches were visible a median 16 min before sunrise. With the benefit of contrast against the sky, observers on the ground were able to visualize large and small branches slightly earlier. Thus, the larger monkeys, *Ateles* and *Lagothrix* were starting to move about the canopy about the time when humans could visualize branches that could support the monkeys’ weight, while the smaller monkeys, *Pithecia* and *Plecturocebus* waited until later. All colors were visible to human observers near the time small terminal branches were visible in the canopy, a median 17 min before sunrise. At ground level, colors were visible slightly later because ambient light is dimmer at ground level.

**Table 3:** Times when canopy features became visible to human observers.

Observer location	Feature visible	Min before sunrise, median	IQR	Number of observations
Canopy	Large branches	27	9	54
Canopy	Terminal branches	16	6	55
Ground	Large branches	42	15	71
Ground	Terminal branches	33	15	70
Canopy	All colors	17	7	63
Ground	All colors	14	5	66

All colors: The time by which blue, green, yellow, and red were all visible.

In one exceptional case there was a full moon and a clear sky, and large branches were visible from the canopy platform 57 min before sunrise. Such bright moonlight is unusual at Tiputini because there is typically some cloud cover. In July and August of 2013 and 2015, only 15% of nonrainy mornings (8/53) were recorded as having clear skies. Occasional bright moonlit mornings might account for some instances when monkeys appeared to have changed sleeping trees during the night or to have left the sleeping site early (See Methods).

#### First feeding bouts

**Fig 5:**
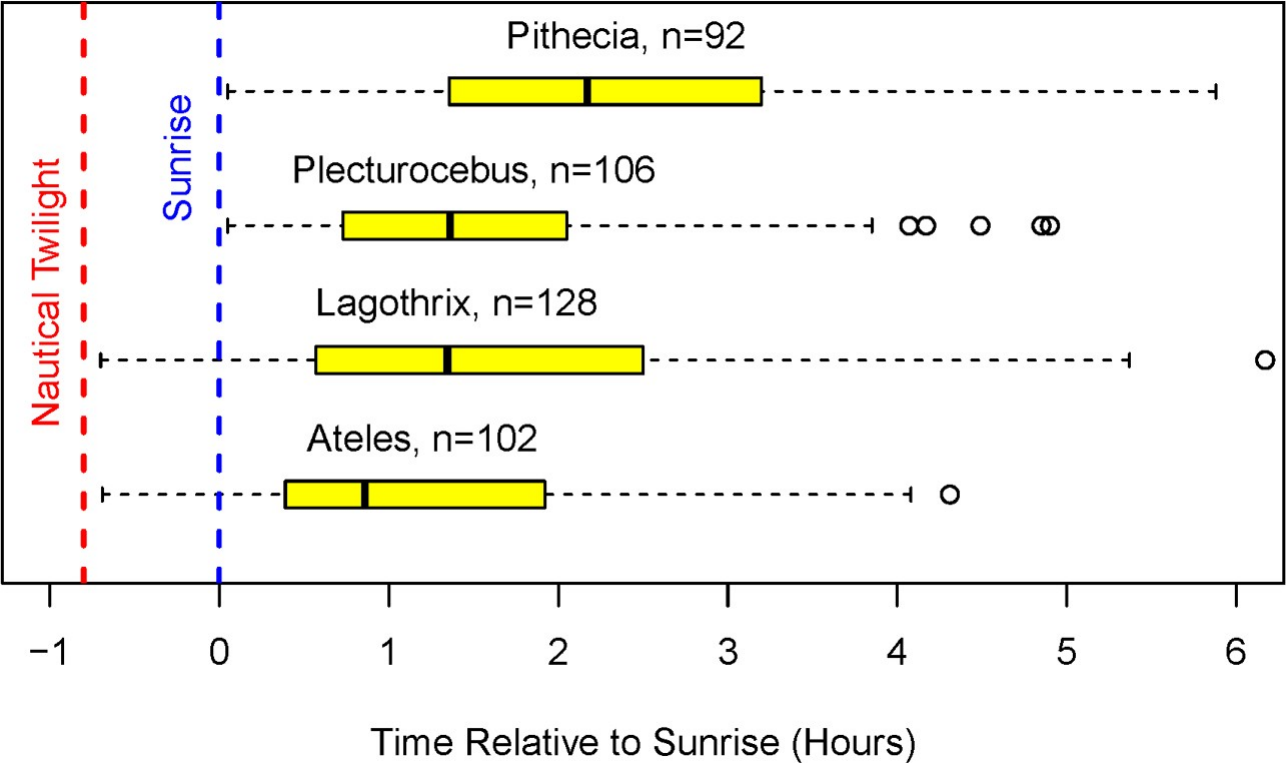
Time to begin first feeding bouts (hours relative to sunrise). Boxplots indicate the median and the interquartile range (IQR); whiskers indicate the extreme values unless there are data points more than 1.5*IQR below the lower quartile or more than 1.5*IQR above the upper quartile, in which case the whiskers stop at the first data points within those boundaries and open circles indicate more extreme points. n = number of observations.

First feeding bouts of each taxon were widely dispersed in time, and many were quite late (Fig 5). *Pithecia* and *Plecturocebus* never had a feeding bout before sunrise. In fact, the median time of *Pithecia’s* first feeding bouts was more than 2 hours after sunrise. Although the larger monkeys sometimes fed as early as a few minutes after the beginning of nautical twilight, the great majority of the feeding bouts of *Lagothrix* (95%) and *Ateles* (91%) also occurred after sunrise.

Fig 5 is based on the total sample of 428 first feeding bouts, but some of the data were recorded at times when one or more of the ecological factors were not documented. For statistical testing of differences among taxa, we used the subset of samples with complete ecological data. Table 4 illustrates that the means and medians for the times of first feeding bouts with complete ecological data were representative of the total sample. The statistical model (Model 2) was based on transformed data and it reduced to a mixed model with year and month as random factors; it accounted for 25% of the variance in times of first feeding bouts across the sample. *Pithecia* fed at least 0.8 h later than the other three taxa (p<0.001), but no other comparisons were statistically significant (all p≥0.9).

**Table 4:** Time of first feeding bouts (hours relative to sunrise).

Taxon	Total Samples	Median (IQR)	Mean ± SE	Complete Samples	Median (IQR)	Mean ±SE
*Pithecia*	92	2.17 (1.84)	2.32 ± 0.14	66	2.21 (1.61)	2.25 ± 0.15
*Plecturocebus*	106	1.36 (1.32)	1.62 ± 0.11	80	1.34 (1.04)	1.54 ± 0.11
*Lagothrix*	128	1.35 (1.93)	1.62 ± 0.12	103	1.06 (1.77)	1.61 ± 0.14
*Ateles*	102	0.86 (1.53)	1.26 ± 0.12	54	0.87 (1.40)	1.23 ± 0.16

Left columns: Number of observations, medians, and means for the the total sample. Right columns: Medians and means for the subset of observations that included data for all three ecological variables—temperature, rainfall, and ripe fruit abundance.

### Delay and distance between the sleeping tree and the first feeding bout

We were interested to learn whether the relatively late feeding times and differences among taxa were due to monkeys choosing to leave the sleeping trees late, or due to long times or distances traveling to the feeding tree. Our stringent criteria (requiring complete ecological data, time of departure from the sleeping tree, and time of first feeding bout, all on the same day) resulted in a reduced number of observations (Table 5), but several outcomes are still clear. The feeding delays represent only 20% (*Ateles*), 39% (*Lagothrix*), 27% (*Plecturocebus*), and 6% (*Pithecia*) of the median times after sunrise before feeding began. When *Ateles* was the reference taxon in the model (Model 3, which reduced from a mixed model to a linear model), its feeding delay was reported as significantly briefer than *Lagothrix* (p = 0.027), but not the other taxa (both p >0.2). In addition, *Ateles’* feeding delays were less dispersed than those of *Lagothrix* (p<0.01) and, likely, *Plecturocebus* (p = 0.06; two-sample Kolmogorov-Smirnov tests). The significant differences in feeding patterns between *Ateles* and *Lagothrix* are consistent with the impressions of observers who have personally studied both taxa at this study site. Other pairwise comparisons between taxa did not detect statistically significant differences (all p >0.2), but that outcome may be due to the limited sample and to statistical adjustments for multiple comparisons.

**Table 5:** Feeding delays and distance traveled.

Taxon	# Records	Median Feeding Delay (h)	Median Distance Traveled (m)	# Records
*Pithecia*	13	0.15	34.2	8
*Plecturocebus*	14	0.37	30.8	8
*Lagothrix*	27	0.52	29.7	16
*Ateles*	20	0.17	76.0	13

Delay between leaving the sleeping tree and time of first feeding bout, and distance from the sleeping site to the feeding tree (medians).

For a subset of the data, we were able to calculate the distance traveled between the sleeping site and the first feeding tree (Table 5). The median distances traveled by the different taxa do not explain the differences in feeding delays or feeding times. For example, *Pithecia* and *Ateles* have nearly identical median feeding delays, but *Ateles* travels more than twice as far to its first feeding tree. And *Lagothrix* has a longer feeding delay than *Ateles*, but it travels less than half as far. Because of the small number of samples, we did not construct statistical models for the comparisons of travel distances.

### Ecological factors: Relationship to departure from the sleeping tree and initiation of feeding

The association of departure time from the sleeping tree with ecological factors was explored for each taxon individually (Models 4–7). We found that: a) lower temperature was significantly associated with later departure by *Ateles* (p<0.01), b) rainfall was significantly related to later departure for *Pithecia* (p<0.01) and perhaps for *Lagothrix* (p = 0.09), and c) *Ateles* tended to depart earlier when ripe fruit was more abundant (p = 0.07). This pattern is consistent with the relationships of ecological factors to timing of first feeding bouts (Table 6); with a larger sample, additional significant relationships consistent with the ecological relationships identified for feeding bouts would likely be recognized.

**Table 6:** Analyses of associations between timing of first feeding bouts and ecological factors.

Taxon andData Format	Ecological Fixed Factors	t value	p value	Records with Complete Data (N) andSkewness Ratio (SR)	% Variance Explained by Fixed Factors
*Ateles* Transformed				N = 54SR = -0.09	24.4%
	Temperature	-3.435	0.002		
	Rainfall	1.650	0.053*		
	Ripe Fruit	-2.848	0.007*		
*Ateles*Raw data				N = 54SR = 0.46	24.1%
	Temperature	-3.136	0.004*		
	Rainfall	1.264	0.106		
	Ripe Fruit	-2.831	0.007*		
*Lagothrix*Transformed				N = 103SR = 2.15	13.2%
	Temperature	-0.986	0.327		
	Rainfall	2.367	0.010*		
	Ripe Fruit	2.538	0.013*		
*Lagothrix*Raw data				N = 103SR = 3.91	14.5%
	Temperature	-0.981	0.329		
	Rainfall	2.627	0.005*		
	Ripe Fruit	2.488	0.011*		
*Plecturocebus*Transformed				N = 80SR = 2.6	10.9%
	Temperature	-1.182	0.241		
	Rainfall	2.771	0.004*		
	Ripe Fruit	-0.997	0.321		
*Plecturocebus*Raw data				N = 80SR = 3.81	10.3%
	Temperature	-0.883	0.380		
	Rainfall	2.811	0.003*		
	Ripe Fruit	-0.813	0.419		
*Pithecia*Transformed				N = 66SR = -1.32	7.3%
	Temperature	-1.91	0.061		
	Rainfall	1.440	0.080		
	Ripe Fruit	-0.154	0.886		
*Pithecia*Raw data				N = 66SR = -0.20	8.8%
	Temperature	-1.908	0.061		
	Rainfall	1.758	0.042*		
	Ripe Fruit	-0.244	0.808		

Analyses are based on models 8–11 of Table 1. For these analyses, random factors did not have significant effects and were removed, except for *Ateles*, where focal animal remained as a significant factor. Column 5: N = number of samples in each model with complete data for all three ecological factors; (SR) = skewness ratio of the residuals of the model. Column 6: Percentage of the variance in feeding bout times accounted for by the ecological fixed factors.

*Indicates p≤0.05.

**Fig 6:**
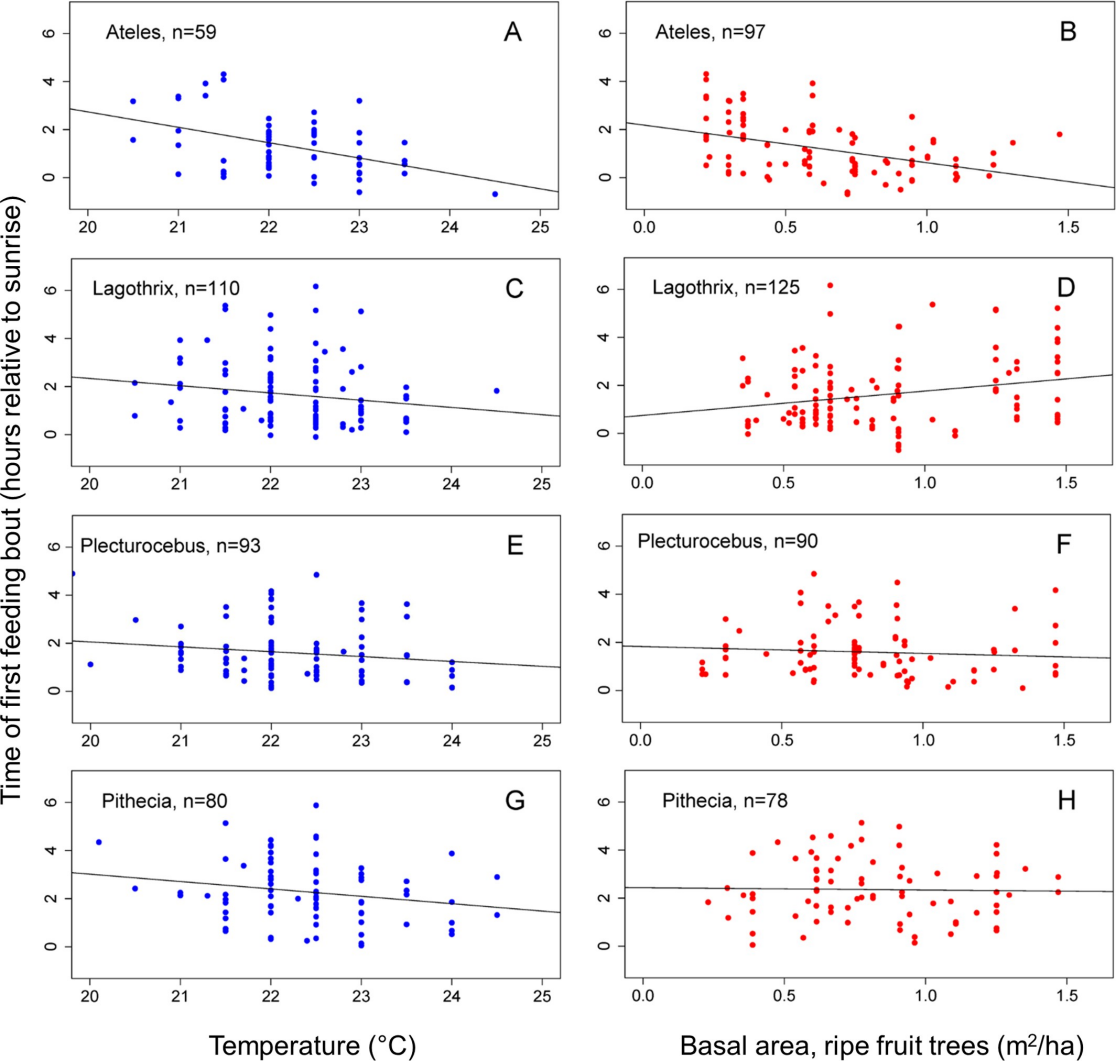
Relationships between times of first feeding bouts and ecological factors. Panels A, C, E, G: Time of first feeding bout as a function of minimum temperature on the day of the observation. Panels B, D, F, H: Time of first feeding bout as a function of ripe fruit abundance in the biweekly period containing the observation day. Least squares regression lines are plotted, based on the displayed points.

We also examined the relationships between times of first feeding bouts and ecological factors for each taxon individually. Statistical outcomes differed only slightly between models based on raw or transformed data (Table 6), which justified using raw data to display relationships (Fig 6) and to estimate quantitatively the effects of ecological factors in easily interpretable units. Note that the numbers of samples listed in Table 6 for use in the statistical models include only those with data for all three ecological factors, whereas the graphs in the figures include larger numbers of observations because they include cases that had missing data for one or two of the factors. For example, there were 54 records of first feeding bouts of *Ateles* that had data for all three factors, but there were 97 that had data for ripe fruit availability and 59 that had data for morning temperature. We felt it appropriate to include all available data for the particular factor (temperature or fruit) represented in each graph because it utilizes the full data set.

#### Temperature

Of the ecological factors, temperature was the one best synchronized with behavior, because it was measured continuously during the observation period. *Ateles* fed earlier (p = 0.002) when temperatures were warmer (Fig 6A). The magnitude of the effect, with other factors held constant, was estimated from the regression coefficient of temperature in the model based on raw data. *Ateles’* first feeding bouts occurred earlier by 0.44 h (26 min) per 1°C increase in the minimum temperature of the day. Although other taxa trended toward earlier feeding with warmer temperatures, *Pithecia* was the only other taxon where the relationship between temperature and the time of the first feeding bout came close to statistical significance (p = 0.061, Fig 6G), with an estimate of 0.35 h (21 min) earlier feeding times per 1°C increase in temperature.

#### Ripe fruit abundance

The relationship of ripe fruit abundance to first feeding times varied among taxa. *Ateles* fed earlier when ripe fruit was more abundant (p = 0.007, Fig 6B), but *Lagothrix* fed later (p = 0.013, Fig 6D). We estimate that, with other factors held constant, *Ateles* first feeding bout occurred *earlier* by 1.1 h per unit of fruit abundance (basal area of trees bearing ripe fruit in m^2^/ha). In contrast, *Lagothrix’s* first feeding bout occurred 0.95 h *later* per unit of fruit abundance. When *Ateles* and *Lagothrix* were included in a model with a *taxon* by *ripe fruit abundance* interaction (Model 12), the difference in the relationship to ripe fruit abundance between the two taxa was statistically significant (p = 0.003). Times of first feeding bouts of *Plecturocebus* (Fig 6F) and *Pithecia* (Fig 3H) were not significantly related to ripe fruit abundance.

#### Rainfall

Because we cannot say what fraction of the 24 h rainfall accumulation occurred in the early morning hours, we did not try to make quantitative estimates of its effects. Nevertheless, the direction of the effect was clear; later times of first feeding bouts were positively related to rainfall (all taxa, p≤0.05). Furthermore, mean rainfall on days when feeding bouts were recorded for *Ateles* was only about half the amounts that were recorded when other taxa were observed, so the effect of rainfall on *Ateles* is likely underestimated. For *Pithecia*, our conclusion of a statistically significant effect of rainfall is based on the model using raw data, which produced a lower skewness ratio than the transformed data and was more consistent with the assumptions of the models.

#### Male-female comparisons

Individual group members of *Ateles* and *Lagothrix* were sufficiently independent in their activity that first feeding bouts of males and females could be separately recorded. For *Ateles*, 35 first feeding bouts of 10 females and 23 first feeding bouts of 8 males were recorded along with complete ecological data. The mean time of first feeding bouts of males was only 2 min earlier than the mean time for females (Model 13, p = 0.997). For *Lagothrix*, 37 first feeding bouts of 20 females, and 17 first feeding bouts of 14 males were recorded along with complete ecological data. Mean times of first feeding bouts of males were 16 min later than mean feeding times of females, but, as for *Ateles*, this difference was not statistically significant (Model 14, p = 0.65).The smaller monkeys, *Plecturocebus* and *Pithecia*, typically foraged in small groups where males and females often fed in close spatial and temporal proximity, and we could not distinguish feeding patterns of males and females.

### Variation in first feeding times throughout the year

One contributor to the large temporal dispersion of feeding bouts was variation throughout the year. To analyze first feeding times over the course of the year, we first grouped the data into bimonthly periods. All taxa tended to start feeding latest in the July-August period, with at least an hour difference between the median for July-August and the bimonthly period with the earliest median first feeding bout time (January-February for *Ateles*, *Plecturocebus* and *Pithecia*; March-April for *Lagothrix*). The largest variation was shown by *Ateles*, where the difference between median times of first feeding bouts in January-February and in July-August was 1.84 h.

**Fig 7:**
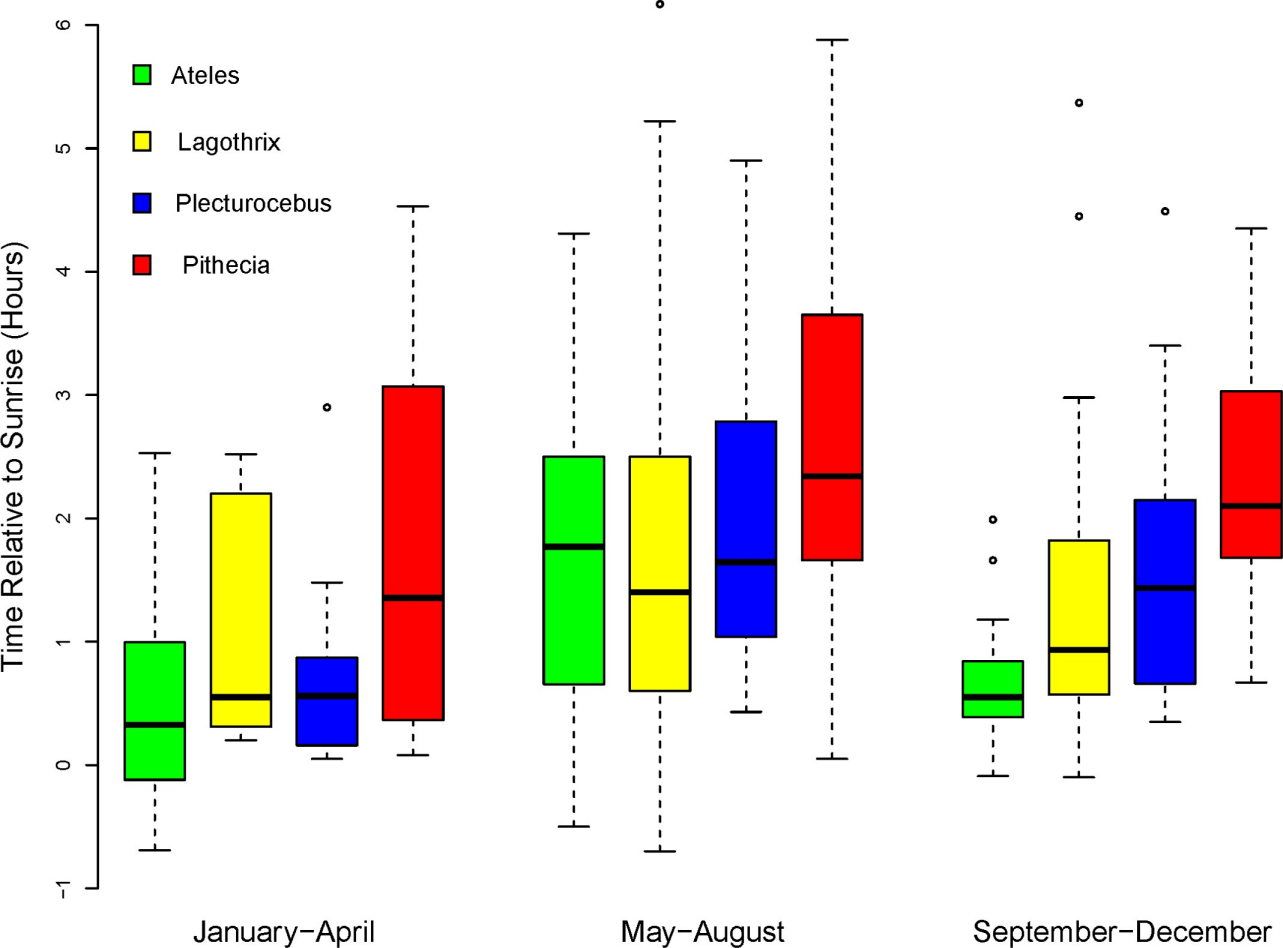
Times of first feeding bouts of all taxa relative to sunrise, grouped by quadrimester. Median values for times of first feeding bouts and numbers of observations are in Table 7.

Because some of the bimonthly groups had relatively small sample sizes we combined the data into quadrimesters (four-month periods) for statistical analyses. All taxa had first feeding bouts earliest in January-April, latest in May-August, and intermediate in September-December (Fig 7). The variation in times of first feeding bouts throughout the year was statistically significant as tested by a one-way ANOVA whether based on raw or transformed data (*Ateles* and *Plecturocebus*, p<0.001; Pithecia, p<0.05). The variation over the year in *Lagothrix*’s feeding times was not statistically significant (p>0.5), but the January-April quadrimester was probably not adequately sampled because only 5 observations were made in that time period (Table 7).

**Table 7:** Median times of first feeding bouts (hours relative to sunrise).

Taxon	January-April	May-August	September-December
*Ateles*	0.33 (n = 20)	1.77 (n = 59)	0.55 (n = 23)
*Lagothrix*	0.55 (n = 5)	1.40 (n = 101)	0.94 (n = 22)
*Plecturocebus*	0.56 (n = 22)	1.65 (n = 68)	1.44 (n = 16)
*Pithecia*	1.36 (n = 20)	2.34 (n = 55)	2.10 (n = 17)

n = number of observations.

#### Quadrimester-by-Quadrimester comparisons

For statistical testing of differences throughout the year we used a model that included a taxon-by-quadrimester interaction term, which allowed us to test which of the differences between taxa and between quadrimesters were statistically significant, while controlling for the effects of the ecological variables. The initial full mixed model (Model 15) reduced to a linear regression model with only fixed factors.

*Pithecia* fed significantly later than *Ateles* in all quadrimesters and later than *Lagothrix* in May-August and September-December (p<0.05); the small sample size for *Lagothrix* in January-February precludes interpretation for that quadrimester. *Pithecia* also fed significantly later than *Plecturocebus* in May-August (p<0.01), and trended later in January-April (p = 0.09). No other comparisons between taxa were statistically significant (all p ≥ 0.4). These results show that *Pithecia’s* tendency to feed later than other taxa occurs throughout the year.

*Ateles* and *Plecturocebus* showed the largest seasonal variation in the timing of first feeding bouts. Both fed significantly later in May-August than January-April (*Ateles*, p<0.01; *Plecturocebus*, p<0.05). *Ateles* fed somewhat later in May-August than September-December (p = 0.07), and *Plecturocebus* fed somewhat later in September-December than January-April (p = 0.08). This seasonality of *Ateles* and *Plecturocebus* is evident even though we have controlled for the measured ecological variables, which suggests the influence of some additional seasonal factor(s). There were no other differences between quadrimesters that approached statistical significance (all p≥ 0.2), including all of the quadrimester comparisons for *Lagothrix* and *Pithecia*.

Regarding *Plecturocebus*, we note that median rainfall on observation days in May-August was 2.1 times as high as rainfall in January-April, and 1.4 times as high as September-December. Such a rainfall pattern could lead to later feeding times in May-August than the average rainfall distribution (Fig 3), implying that the numbers in Table 7 and Fig 7 may represent an overestimate of the magnitude of *Plecturocebus’* usual seasonal variation.

## Discussion and conclusions

### Similarities in the temporal sequences of morning activities of all taxa

The daily temporal patterns of all taxa shared two basic features: 1) these diurnal monkeys only began their activities after the onset of nautical twilight (Fig 4), and 2) 90 to 100% of their first foraging bouts occurred after sunrise (Fig 5). This pattern occurred in spite of the fact that, among these four species, all males and many females are dichromats [55], and might, theoretically, take advantage of dim light during the early morning period. Overall, these results suggest that any dim-light advantage that dichromatic individuals might possess is not systematically exploited in the early morning period.

Data from animals at our study site show polymorphic M/L opsins in all four taxa, with at least one-fourth of the females in each taxon possessing trichromatic color vision [55, 56]. Nevertheless, both males and females typically waited until after sunrise to feed, long after the time when trichromatic human observers had sufficient ambient light to detect small food items and perceive colors. Assuming that the monkeys have visual capabilities similar to humans, they do not appear to be feeding as early as ambient light would permit.

It is relevant to note that our analyses do not formally take into account differences in light levels among forest strata, because these differences are expected to be small compared to the changes in light levels around the time of sunrise. We have compared illuminance at the top of the canopy and at ground level in dense forest and the maximum attenuation of light at ground level is 2 log units (powers of ten). However, the attenuation of light in the forest where the monkeys are active is even less, because these taxa do not come to the ground to forage, and they sleep in tree crowns. In contrast, depending on cloud cover, the range of illuminance between the onset of nautical twilight and 30 min after sunrise is about 5–7 log units, or 3–5 orders of magnitude greater than differences seen among forest strata (D.M. Snodderly, unpublished data). Thus, the attenuation of light within the forest is expected to play only a minor role in the timing of the behaviors we have studied.

### Evidence for temporal niche partitioning in the morning

Although there is substantial overlap in the morning activities of the monkeys, there are at least two distinct differences that can be considered components of temporal niche partitioning:

1. The larger, more frugivorous monkeys, *Ateles* and *Lagothrix*, leave their sleeping trees and begin to forage earlier than the smaller monkeys. For *Ateles* and *Pithecia* especially, the relatively short delay between leaving the sleeping tree and the time of first feeding bouts imply that the monkeys’ decision to leave the sleeping tree was the major determinant of the time of first feeding bouts. An early start may be related to exploitation competition for the limited resource of ripe fruit [57].
2. *Pithecia*, a seed predator, begins to feed later than the other three taxa. This late start may indicate lower competition achieved by specialized feeding on non-preferred tough fruits and seeds.

### Factors related to species differences in the timing of morning activities

Some of the key characteristics and differences among the primate taxa at Tiputini emerging from the analyses are summarized briefly in Table 8 to help structure the remainder of the Discussion.

**Table 8:** Relationships of first movements and feeding times to biotic and abiotic factors.

Taxon	Body Weight (kg)	Depart Sleep Tree	First Feed Bout	Temperature Sensitivity	Fruit Abundance	Seasonal Variation
*Ateles*	9.0 F—9.3 M	-0.07	0.86	-0.44	-1.09	high
*Lagothrix*	5.5 F—7.5 M	0.08	1.35	—	0.95	indeterminate
*Plecturocebus*	0.8 F—0.9 M	1.28	1.36	—	—	high
*Pithecia*	2.0 F—2.6 M	1.44	2.17	-0.35	—	low

Body Weight: Mean weights of females (F) and males (M). *Ateles*, F, n = 5, M, n = 3; *Lagothrix*, F, n = 5, M, n = 5; *Plecturocebus*, F, n = 5, M, n = 4; *Pithecia*, F, n = 2; M, n = 4. Depart Sleep Tree: Median time of departure from the sleeping tree in hours relative to sunrise. First Feed Bout: Median time of first feeding bout in hours relative to sunrise. Temperature Sensitivity: Change in time of first feeding bout in hours/°C. Fruit Abundance: Change in time of first feeding bout in hours per unit of fruit abundance.

—indicates no statistically significant relationship.

### Body size: energetic needs and predation risk

Some of the differences we observed among taxa appear to be linked to body size. The earlier start of the larger monkeys may reflect greater energetic needs. For *Ateles*, the short retention time of their digestive system [30] could also motivate them to begin foraging early after an overnight period of fasting.

The smaller taxa are potential prey for a large range of predators, and predation risks could influence the start of morning activities. There is an intact predator community at Tiputini [34], and predation attempts by raptors and tayras—both diurnal—on the smaller monkeys have been documented, [41, 58]. However, for predation to delay morning activity, one would have to assume that the predators are nocturnal or active in dim light, thus driving the monkeys to wait until full daylight when predators are less active. Although some nocturnal predators, such as snakes [58, 59] or potentially felids, might exert this pressure, the most frequent predators of primates in the Neotropics are thought to be raptors [60], which have relatively poor vision in dim light [61]. Consistent with their visual specializations, we are not aware of any evidence for raptors that hunt monkeys in the twilight period. Thus, it seems unlikely that predation pressure could account for the late start of morning activities of the small monkeys.

### Temperature and thermoregulation

The later start of the smaller monkeys could also be related to thermoregulation, which is thought to be of particular importance for smaller animals [62]. The pre-dawn hours are the coolest time of day, and the smaller monkeys could wait for direct sunlight or warmer temperatures that follow sunrise [63, 64]. However, our results do not adhere to the expected pattern of greater effects of temperature on smaller animals. The time of first feeding by the smallest monkey of the group, *Plecturocebus*, was not significantly related to temperature. The effects of temperature on *Plecturocebus* may be buffered by sleeping huddled in contact with other group members, often in trees with leafy vine tangles that may offer some protection from wind or heat loss (*cf*. [63, 65–68]).

Contrary to expectations, the largest monkey, *Ateles*, was the taxon whose onset of activity and timing of first feeding bouts was most strongly affected by temperature (Table 6, Fig 6). This outcome may reflect the unusual anatomical proportions of *Ateles*, which has extremely long limbs, a long tail, and a compact torso. *Ateles’* body shape may have a higher than expected surface-to-volume ratio for its weight that would cause heat loss, in addition to requiring more warmth for muscular flexibility and efficiency. Furthermore, *Ateles* tends to sleep in relatively exposed locations not in contact with other individuals [69] and hence could be subject to significant heat loss during the night. *Pithecia* also does not huddle, and usually sleeps out-of-contact with group-mates (unpublished observations), which may help to explain why the sakis tend to delay activity in cooler conditions. It seems likely that the contrasting pattern of temperature sensitivity among species is related to social grouping as least as much as it is related to anatomical characteristics. In a larger context, as global warming proceeds, *Ateles* may begin to forage and to feed even earlier, which could separate it further from the less sensitive species, *Lagothrix* and *Plecturocebus*, resulting in a more differentiated temporal partitioning of the morning.

Other accounts of effects of temperature on activity patterns of large wild primates primarily consider terrestrial or semi-terrestrial species and focus either on avoidance of overheating (e.g. [70–72] or coping with extreme cold [73]. Among large arboreal primates, gibbons have been shown to reduce their activity during cold weather in montane regions, where minimum temperatures are as low as 0ºC [74]. However, our finding of temperature sensitivity in a large arboreal primate where daily minimum temperatures only ranged from 20.5–24.5 ºC suggests that moderate changes in temperature may have stronger effects on primate activity patterns than generally appreciated; this realization should be incorporated into discussions of climate change [75].

### Effects of rainfall

As noted by others [76], a very small amount of rain (< 1 mm) can suppress activity of primates when it occurs at the time the behavior would normally occur. This behavioral response is presumably a component of thermoregulation since rainwater immediately absorbs heat from the body, and evaporation continues the heat loss. It may also represent a response to poor visibility and slippery substrates.

### Ripe fruit abundance and dietary preferences

The influence of ripe fruit abundance on the time to initiate feeding reflects the dietary specializations of the different taxa. The most dramatic comparison is between *Ateles* and *Pithecia*. *Ateles* is the most frugivorous taxon, relying heavily on ripe fruit, [3, 77], while *Pithecia* belongs to a genus of seed predators that feed preferentially on unripe and hard-to-process fruits. *Ateles* was the earliest to feed (Fig 5), and it fed more quickly when ripe fruit was more readily available (Fig 6), whereas *Pithecia* was last to feed, and fruit abundance had no effect on timing of its first feeding bouts (Fig 6, Table 6). Thus, *Ateles* and *Pithecia* represent dietary extremes that correspond to temporal extremes in morning activity, feeding behavior, and in niche separation.

Ripe fruit is a transient resource that is consumed by a wide variety of frugivores, including primates, birds, squirrels, and bats. As the most frugivorous primate taxon, *Ateles* should face strong competition for this preferred resource [78, 79], and feeding early could confer an advantage. A recent analysis of early morning foraging by wild chimpanzees (*Pan troglodytes verus*), who are highly frugivorous, advanced a similar viewpoint [80]. Chimpanzees left their sleeping trees to feed earlier when briefly ripening figs and small fruits were available compared to the times when they fed on other food sources. This behavior is consistent with the hypothesis that dependence on ripe fruit leads to scramble competition, which pushes frugivores to start feeding earlier and contributes to temporal niche partitioning.

However, this line of reasoning does not explain why *Ateles* should wait so long to feed when fruit abundance is low. The late start may imply a foraging strategy of energy conservation during periods of low food abundance [81] or low temperature, wherein animals reduce physical activity instead of increasing foraging time to maintain a desired caloric intake. A similar strategy has been proposed for gibbons (*Nomascus concolor*) in China [74].

*Lagothrix* utilizes non-fruit foods more than its close relative, *Ateles*, but is still considered highly frugivorous [3]. However, we found that *Lagothrix* feeds later as more ripe fruit is available, rather than earlier like *Ateles* (Fig 6B and 6D). This difference between these two closely related taxa may help them to miminize competition and share the environment. The difference may also be related to availability of other items in *Lagothrix’s* diet, such as animal prey, especially insects [3, 29, 39].

*Plecturocebus* has a varied diet ([31, 82], and unpublished data), and the timing of its first feeding bouts is not significantly related to our measures of ripe fruit abundance. However, this relationship should be reexamined when measures of abundance of fruits preferentially consumed by *Plecturocebus* are available (*cf*. [29]) and the abundance of non-fruit dietary sources are better known. In other environments, *Plecturocebus* species increase intake of seeds or leaves in periods of low fruit abundance, making them less dependent on fruit [83, 84].

### The seasonal cycle of first feeding times

For all taxa, the timing of first feeding bouts followed a similar annual pattern, with the latest times occurring in the May-August quadrimester (Fig 7). Seasonal variations in our measured ecological factors contribute to the seasonal variation in feeding times, but do not fully explain it.

Early morning temperature and ripe fruit abundance had a consistent temporal pattern. Morning temperatures and ripe fruit abundance on observation days were lowest in the May-August quadrimester, as would be expected from the local ecology (Figs. 2 and 3). For *Ateles*, low values of temperature and ripe fruit abundance were both associated with delayed feeding, and the combined effect, along with unknown factors, contributed to its large seasonal variation in feeding times.

The abundance of ripe fruit is widely considered to be a critical seasonal influence on primate activity patterns [7, 85], but it was associated with initiation of feeding in *Ateles* and *Lagothrix* in opposite ways. For *Lagothrix*, low abundance of ripe fruit was associated with earlier feeding, with the net result being that *Lagothrix*’s annual pattern was less pronounced. Our measure of ripe fruit abundance had no explanatory power for *Plecturocebus* or *Pithecia*.

A key question that remains is whether the monkeys, particularly *Ateles*, compensate for the later start in the May-August period by extending their day and continuing to forage later before entering the sleeping tree. If they do not extend their day, their behavior would be consistent with an interpretation that they are conserving energy in a period of low resources by reducing activity, rather than expending energy to maintain a relatively stable caloric intake.

The study site at Tiputini is a very rich equatorial habitat without a pronounced dry season, but it still has moderate seasonal variation of important environmental factors. Our future analyses will include study of the activity patterns of the monkeys at Tiputini over the course of the 24 h diel cycle [86–88] to test hypotheses about their temporal partitioning of the day and strategies for coping with environmental variation and resource seasonality. We are also characterizing the photopigment opsins of the monkeys [55] so that we can clarify the relationships between color vision phenotype, feeding ecology, and niche partitioning.

## Supporting information

S1 Table Dataset used for the study. *Group or Individual*: For *Plectrocebus* and *Pithecia*, social group is indicated by one or two letters, and both sexes are considered together. For *Ateles* and *Lagothrix*, the individual and its sex is indicated. If the individual or sex was unknown, the corresponding cell is empty. When a female/male pair of individuals is listed together, they were engaged in the same activity at the same time. When comparing sexes, each member of the pair was included in the sample for its sex. *Morning Status*: YMA: Monkeys were encountered in the sleeping tree and defecation/urination, time of movement from the sleeping tree, and first feeding bout were all continously recorded in the ‘morning activity’ protocol. YFN: Monkeys were encountered in the sleeping tree and defecation/urination, time of movement from the sleeping tree, and first feeding bout were all continously recorded in the observer’s field notes. Y: Monkeys were encountered in the sleeping tree. Feeding bouts and defecation/urination were continuously recorded in the protocol but movement was not. ME: Monkeys were moving when encountered and had departed the sleeping tree but were still within sight of the sleeping site. FE: Monkeys were feeding when encountered and had departed the sleeping tree but were still within sight of the sleeping site. EY: The sleeping site was not known precisely but monkeys were encountered more than 0.3 hr before sunrise and they were inactive. *Time re Sunrise*: Time in hours relative to sunrise of the first occurrence of the activity. Negative numbers indicate occurrences before sunrise; positive numbers indicate occurrences after sunrise. *Minimum Temp*: Minimum temperature for the day in degrees Centigrade, which occurred in the early morning hours just before sunrise. *24 hr rainfall*: Accumulation in mm of rain over the 24-hour period preceding 6–8 pm of the current day. *Fruit Abundance*: Basal area in m^2^/hectare of trees bearing ripe fruit along the phenology sampling transect. Missing values for temperature and fruit abundance are indicated by NA.
(CSV)

S2 Table Random factors in final models. (DOCX)
