# Supplementary material for: Initiation of feeding by four sympatric Neotropical primates (Ateles belzebuth, Lagothrix lagotricha poeppigii, Plecturocebus (Callicebus) discolor, and Pithecia aequatorialis) in Amazonian Ecuador: Relationships to photic and ecological factors
Source: PLoS One. 2019 Jan 23;14(1):e0210494. doi: 10.1371/journal.pone.0210494 (PMC6344106; doi:10.1371/journal.pone.0210494)
Supplement: S2 Table — (DOCX) [file pone.0210494.s002.docx]

| **Response Variable in Statistical Models of Table 1.** | **Random Factors in Final Model** |
| --- | --- |
| 1) Time to depart sleeping tree | None |
| 2) Time of first feeding bout | Month and Year |
| 3) Delay between departure from sleeping tree and feeding | None |
| 4-7) Time to depart sleeping tree, one model for each taxon | *Ateles, Lagothrix, Plecturocebus,* None; *Pithecia*, Year and Group |
| 8-11) Time of first feeding bout, one model for each taxon | *Ateles*, Animal ID; *Lagothrix*, *Plecturocebus*, *Pithecia*, None. |
| 12) Time of first feeding bout in relation to Ripe Fruit Abundance | Animal ID or Group, Month, and Year |
| 13) Time of Ateles first feeding bout,  Sex differences | None |
| 14) Time of *Lagothrix* first feeding bout,  Sex differences | None |
| 15) Seasonal variation in times of first feeding bouts | None |
